# Idiopathic Calcinosis Cutis Universalis in a Child

**DOI:** 10.7759/cureus.113816

**Published:** 2026-08-02

**Authors:** Oumaima Zouine, Hanane Baybay, Aimane Zaim, FatimaZahra Mernissi

**Affiliations:** 1 Department of Dermatology, Centre Hospitalier Universitaire Hassan II, Fez, MAR

**Keywords:** calcinosis cutis, calcinosis universalis, dermatomyositis juvenile, pediatric dermatology, sodium thiosulfate

## Abstract

Calcinosis cutis is the abnormal deposition of calcium salts in the skin and subcutaneous tissue. In children, it is rare and most often dystrophic, occurring in the setting of an underlying connective tissue disease, with juvenile dermatomyositis being the leading cause. Idiopathic calcinosis cutis universalis, defined by diffuse calcium deposition without preceding tissue damage, metabolic disturbance, or identifiable connective tissue disease, is exceptional in the pediatric population and remains a diagnosis of exclusion. We report the case of a nine-year-old girl with a two-year history of multiple painful subcutaneous calcified lesions that progressively spread over the body and impaired walking. Phosphocalcic balance, parathyroid assessment, and an extensive autoimmune workup, including myositis- and scleroderma-specific antibodies, were unremarkable, and there were no clinical features of dermatomyositis or systemic sclerosis. Whole-body computed tomography showed diffuse periarticular soft tissue calcifications without bone or visceral involvement. A diagnosis of idiopathic calcinosis cutis universalis was made after the exclusion of dystrophic, metabolic, and iatrogenic causes. Management was multimodal and resulted in marked clinical, functional, and radiological improvement, with healing of fistulized lesions and restoration of mobility. This case highlights both the diagnostic rigor required before labeling pediatric calcinosis as idiopathic and the need for prolonged surveillance, as an underlying connective tissue disease may declare itself later.

## Introduction

Calcinosis cutis refers to the abnormal deposition of calcium salts, predominantly calcium phosphate in the form of hydroxyapatite crystals, within the skin and subcutaneous tissues. It may extend to deeper structures, including fascia, muscles, tendons, and, rarely, visceral organs [1]. Calcinosis cutis represents a heterogeneous group of disorders rather than a single disease entity and is traditionally classified into five major categories: dystrophic, metastatic, iatrogenic, idiopathic, and calciphylaxis [1]. The term “universalis” refers to diffuse and extensive involvement of multiple anatomical sites, in contrast to the more localized form known as calcinosis circumscripta.

In children, calcinosis cutis is uncommon and is most frequently associated with dystrophic mechanisms occurring in the setting of connective tissue diseases. Juvenile dermatomyositis is the most commonly reported underlying condition, with calcinosis occurring in approximately 20% to 75% of affected patients, particularly in severe, refractory, or delayed-diagnosis cases [2]. In contrast, idiopathic calcinosis cutis universalis, defined by diffuse calcium deposition in the absence of previous tissue injury, with normal phosphocalcic metabolism and no identifiable underlying disorder, is exceptionally rare in childhood; only a small number of pediatric cases have been reported in the literature to date [3]. We report a rare case of idiopathic calcinosis cutis universalis in a young girl with extensive painful lesions and significant functional impairment.

## Case presentation

A nine-year-old girl with no relevant personal or family medical history was referred to our department for multiple painful subcutaneous lesions that had progressively evolved over two years. The lesions initially appeared over both knees before rapidly extending to the trunk, elbows, back, and plantar surfaces of the feet, leading to significant pain and impaired walking. She had initially consulted a surgeon for surgical excision before being referred for further diagnostic evaluation. Despite the progressive disease burden, her general condition remained preserved, although she reported unquantified weight loss without fever or other constitutional symptoms.

**Figure 1:**
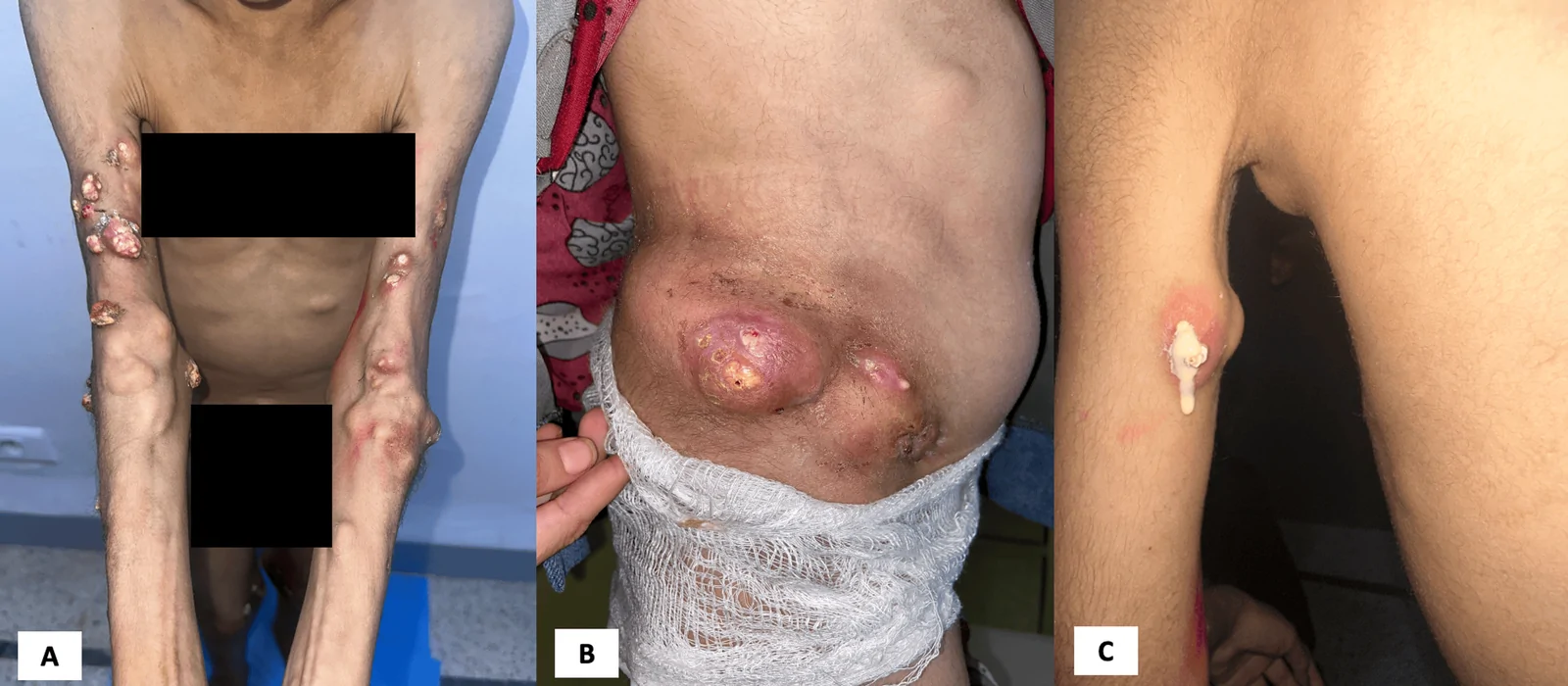
Clinical presentation of extensive calcinosis cutis. (A) Multiple hard subcutaneous calcified nodules and plaques involving the upper limbs, with a predominant periarticular distribution and surrounding erythema and fistulization. (B) Two large fistulizing calcified tumors of the iliac region with an erythematous, crusted surface and transepidermal extrusion of chalky calcareous material. (C) Fistulized calcinosis lesion of the limb with extrusion of chalky white material.

Physical examination revealed multiple hard, stony subcutaneous nodules and plaques of variable sizes, diffusely distributed over the trunk and limbs and firmly adherent to the deep planes. Several lesions showed cutaneous fistulization with spontaneous extrusion of chalky white material, consistent with transepidermal elimination of calcium deposits, and some were surrounded by erythema and inflammatory changes. The gluteal lesions were particularly painful and responsible for major functional impairment, limiting her ability to walk. Two large fistulizing calcified tumors were also noted in the iliac region, with erythematous, crusted surfaces. The largest lesion measured approximately 20 cm along its longest axis (Figure 1).

**Figure 2:**
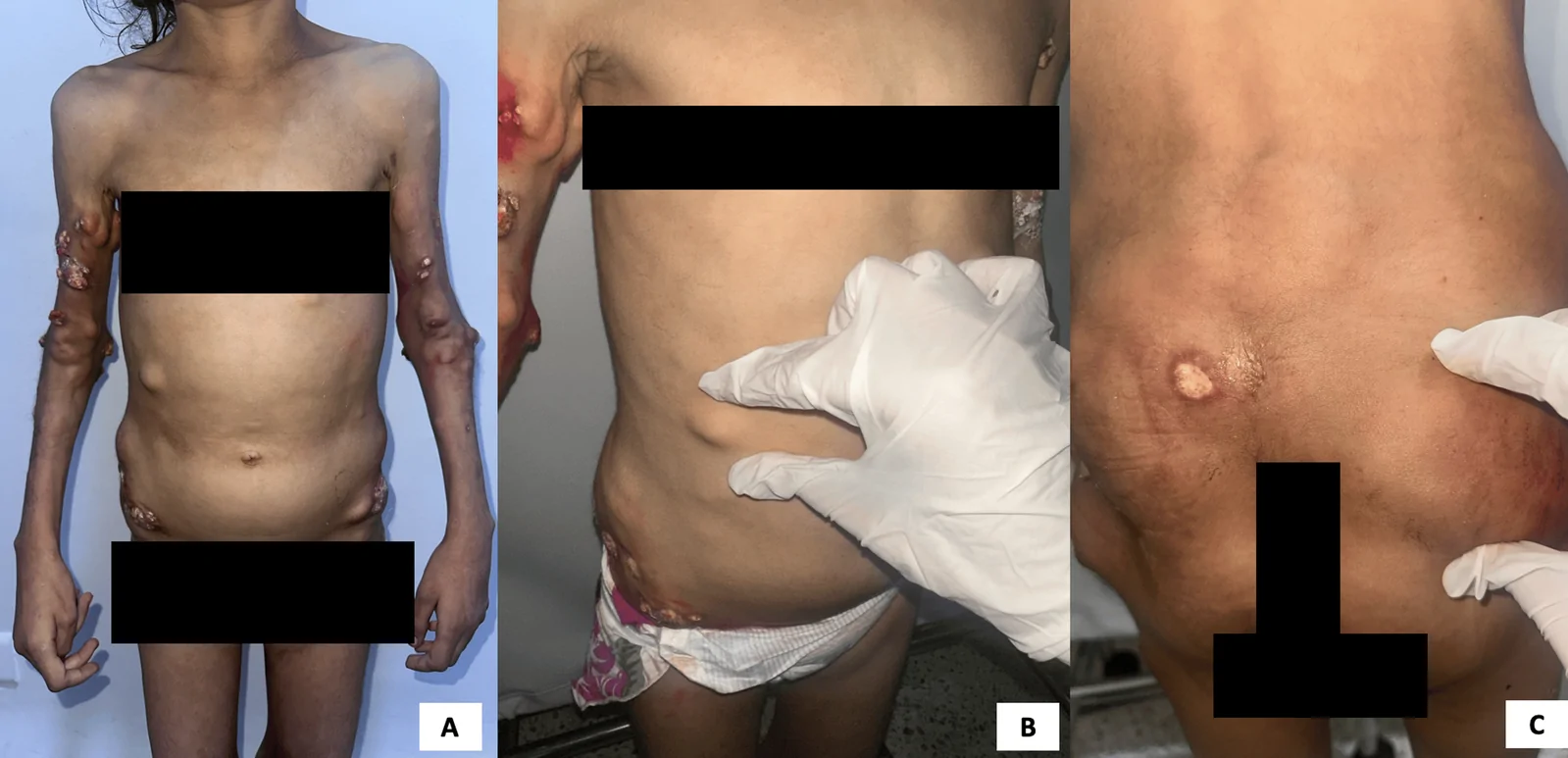
Clinical presentation of calcinosis cutis involving the trunk and lower limbs. (A) Multiple subcutaneous calcified nodules and plaques involving the trunk, abdominal wall, and inguinal folds, with fistulized lesions on the upper limbs and axillary regions. (B) Close-up view of a subcutaneous calcified lesion on the trunk. (C) Close-up view of a large painful gluteal calcified lesion measuring approximately 15 cm.

Further clinical examination demonstrated extensive involvement of multiple anatomical sites, including the trunk, abdominal wall, inguinal folds, axillary regions, upper limbs, and medial thighs, with numerous subcutaneous calcified lesions of variable sizes and consistencies (Figure 2).

There was no proximal muscle weakness, myalgia, dysphagia, arthralgia, arthritis, Raynaud phenomenon, cutaneous sclerosis, digital ulcers, respiratory symptoms, gastrointestinal manifestations, or signs of peripheral vascular ischemia. 

**Figure 3:**
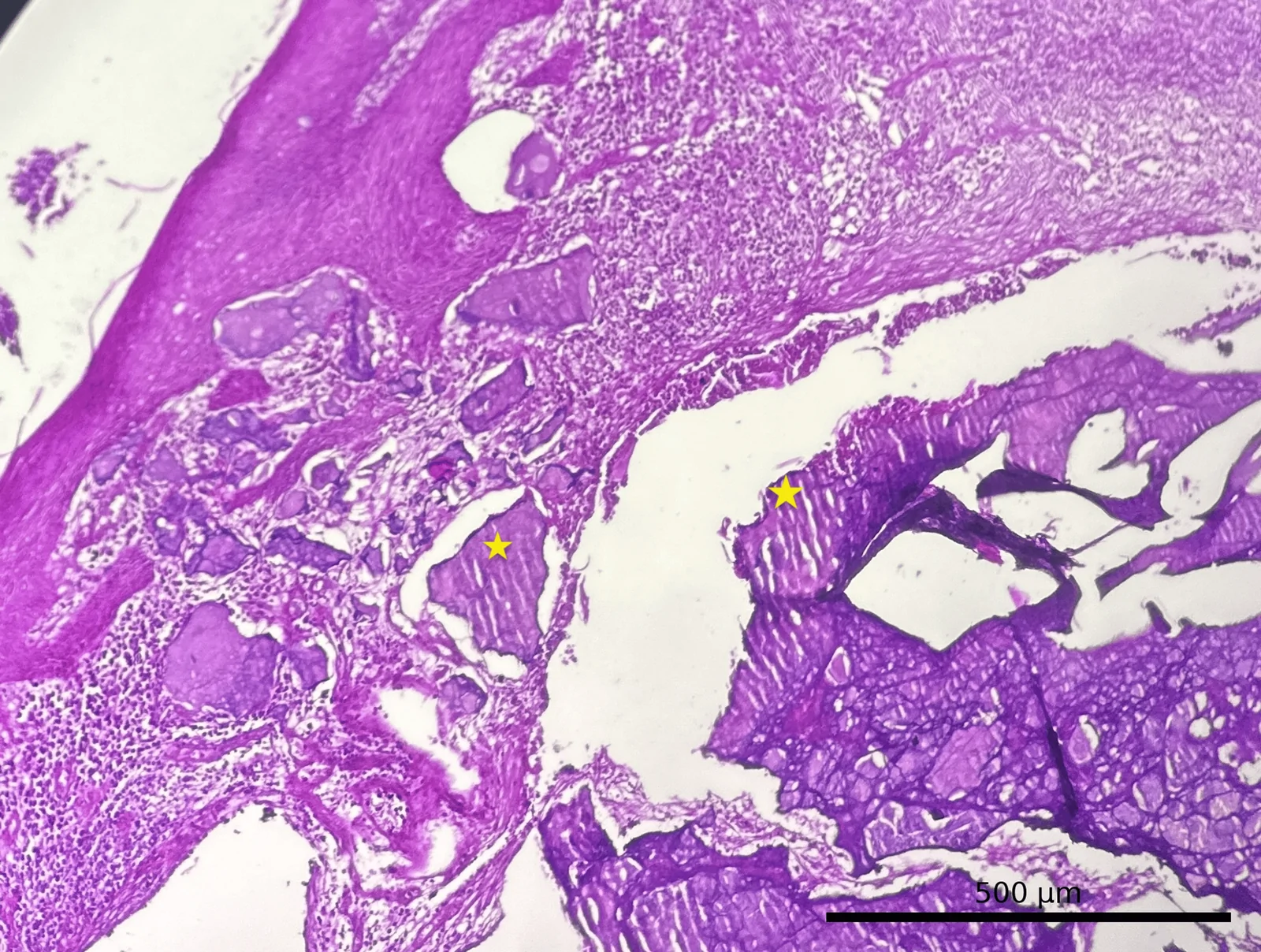
Histopathological examination of the skin biopsy (hematoxylin and eosin). Skin biopsy showing extensive dermal and subcutaneous amorphous calcium deposits (yellow asterisk), confirming the diagnosis of calcinosis cutis. Tissue: skin (dermis and subcutaneous tissue); original magnification ×100. Scale bar = 500 µm.

The diagnosis of calcinosis cutis was clinically evident, given the multiple stony subcutaneous masses with transepidermal extrusion of chalky calcareous material. A skin biopsy was therefore performed mainly to support the exclusion of relevant differential diagnoses. Histopathological examination showed extensive dermal and subcutaneous deposits of amorphous basophilic calcified material with a characteristic fractured appearance, surrounded by a discrete inflammatory reaction without a multinucleated giant cell reaction. The diagnosis was made using hematoxylin and eosin staining, which clearly demonstrated the calcium deposits. A von Kossa stain was not performed because it was not considered necessary, given the typical morphology (Figure 3).

Laboratory investigations revealed microcytic hypochromic anemia associated with a marked inflammatory syndrome, including inflammatory changes on serum protein electrophoresis. Serum creatine kinase was normal, whereas lactate dehydrogenase was mildly elevated. To exclude metastatic calcification secondary to disorders of calcium-phosphate metabolism, serum calcium, phosphorus, parathyroid hormone, renal and liver function tests, and serum and urinary electrolytes were assessed and found to be within normal limits, whereas vitamin D deficiency was noted. Because chronic infection was considered among the differential diagnoses in view of the marked inflammatory syndrome, a tuberculosis workup, including QuantiFERON-TB Gold testing, was performed and yielded negative results. Detailed laboratory findings are summarized in Table 1. 

**Table 1:** Laboratory investigations. Summary of hematological, inflammatory, phosphocalcic, renal, hepatic, and immunological parameters. Reference ranges correspond to the pediatric values of our laboratory. CK: creatine kinase; LDH: lactate dehydrogenase; PTH: parathyroid hormone; AST: aspartate aminotransferase; ALT: alanine aminotransferase.

Parameter	Patient value	Reference range
Hemoglobin	9.1 g/dL	11.5–15 g/dL
Mean corpuscular volume (MCV)	77 fL	78–88 fL
Mean corpuscular hemoglobin (MCH)	23 pg	24–30 pg
Serum iron	0.2 mg/L	0.6–1.8 mg/L
Erythrocyte sedimentation rate	210 mm (2nd hour)	< 20 mm
C-reactive protein	37 mg/L	< 5 mg/L
Serum protein electrophoresis	Inflammatory profile	Normal profile
Creatine kinase (CK)	30 U/L	0–145 U/L
Lactate dehydrogenase (LDH)	467 U/L	120–300 U/L
Total serum calcium	90 mg/L	88–108 mg/L
Serum phosphorus	59 mg/L	40–70 mg/L
Parathyroid hormone (PTH)	61 pg/mL	15–65 pg/mL
25-hydroxyvitamin D	11.6 ng/mL (initially < 3)	30–100 ng/mL
Serum creatinine	5 mg/L	5–12 mg/L
Blood urea	0.24 g/L	0.11–0.38 g/L
Aspartate aminotransferase (AST)	28 U/L	0–35 U/L
Alanine aminotransferase (ALT)	8 U/L	0–35 U/L
Serum sodium	136 mmol/L	135–145 mmol/L
Serum chloride	104 mmol/L	98–106 mmol/L
Total serum protein	80 g/L	66–83 g/L
QuantiFERON-TB Gold	Negative	Negative

Given the extensive calcinosis and inflammatory syndrome, an extensive autoimmune workup was performed to investigate an underlying connective tissue disease, particularly juvenile dermatomyositis and juvenile systemic sclerosis. Antinuclear antibodies, anti-double-stranded DNA, anti-centromere, anti-Scl-70, rheumatoid factor, anti-mitochondrial, anti-smooth muscle, anti-LKM, anti-SLA antibodies, and a comprehensive myositis-specific antibody panel (anti-NXP2, anti-TIF1-γ, anti-Mi-2, and anti-SAE) were all negative. Complement levels were normal. Because systemic sclerosis remained a diagnostic consideration, pulmonary function testing, upper gastrointestinal endoscopy, and esophageal manometry were performed. Pulmonary function testing demonstrated a mild restrictive ventilatory defect, whereas esophageal investigations showed no evidence of gastrointestinal involvement.

**Figure 4:**
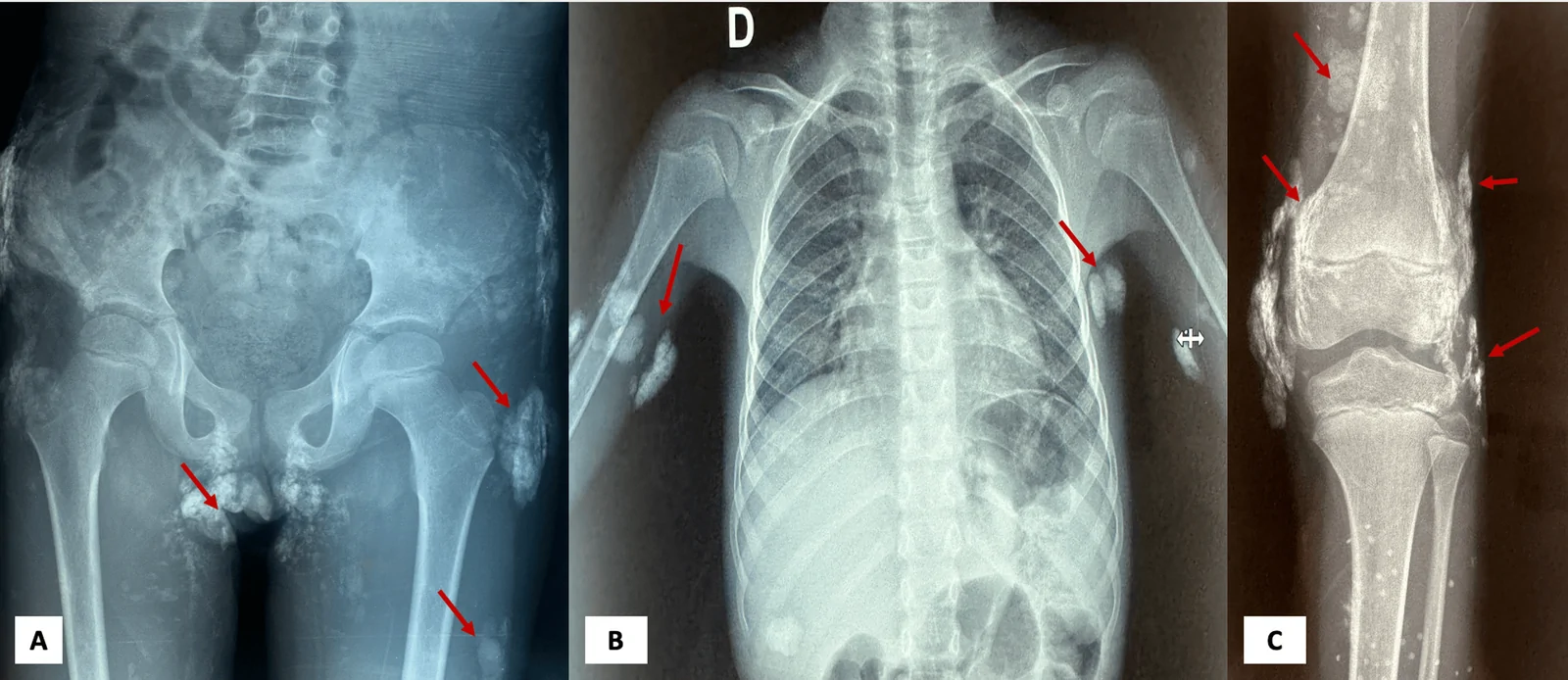
Radiographic appearance of calcinosis cutis. (A) Pelvic radiograph showing extensive, irregular, and lobulated radiopaque calcified deposits within the soft tissues of the gluteal region. (B) Trunk radiograph demonstrating multiple diffuse and multifocal radiopaque calcifications distributed throughout the subcutaneous soft tissues. (C) Knee radiograph showing periarticular soft tissue calcifications with a multilobulated appearance, without evidence of underlying bone involvement. Red arrows indicate the calcinosis lesions in all panels.

**Figure 5:**
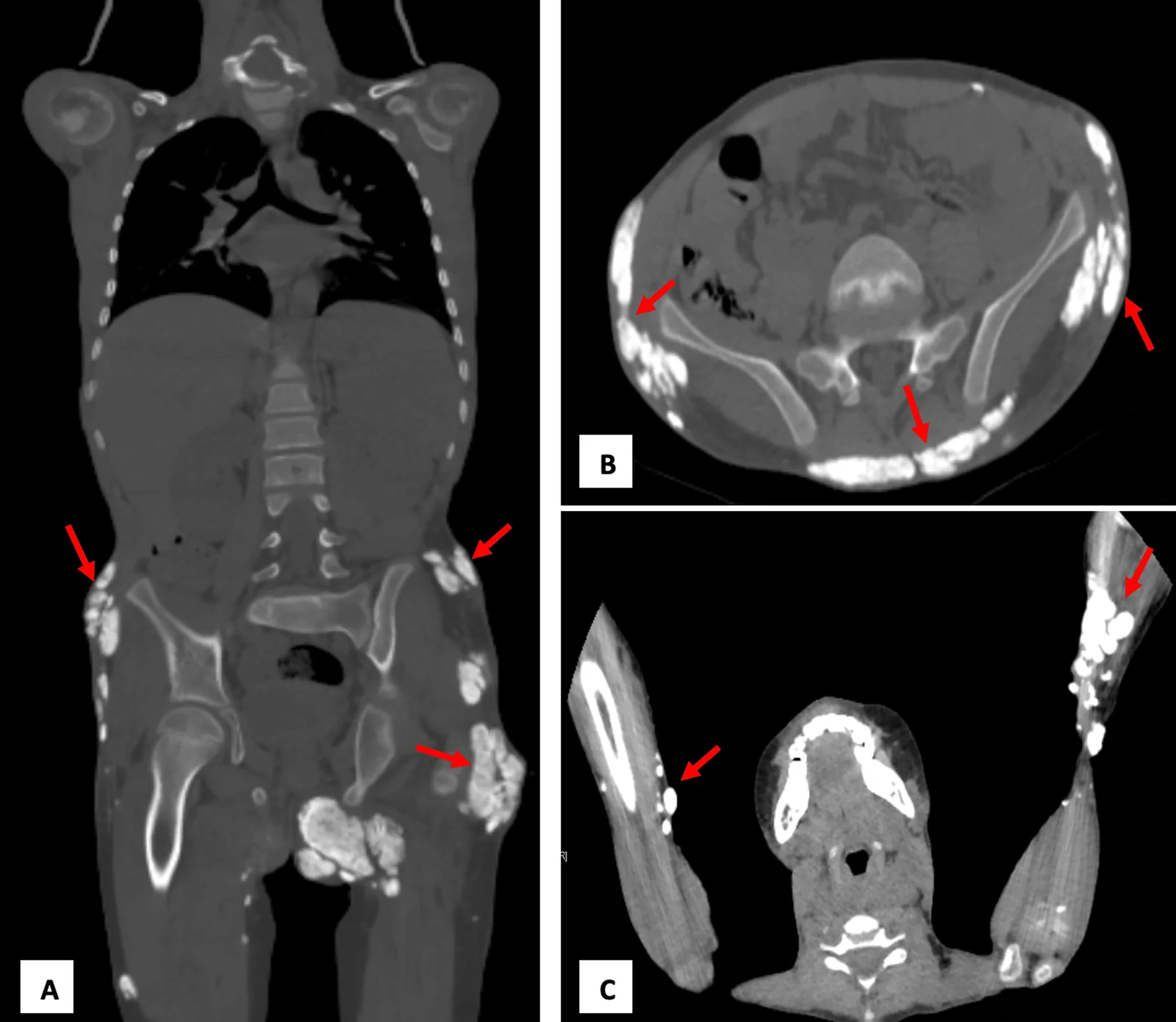
Whole-body computed tomography. (A) Coronal reconstruction showing extensive subcutaneous and periarticular soft tissue calcifications (red arrows) in the pelvic, gluteal, and lower limb regions, without visceral or thoracic involvement. (B) Axial view at the pelvic/inguinal level showing bilateral subcutaneous calcified deposits (red arrows). (C) Axial view at the level of the shoulders showing calcified soft tissue deposits of both proximal upper limbs (red arrows). No bone destruction, lymphadenopathy, or neoplastic lesion was identified.

Imaging evaluation included plain radiographs and whole-body computed tomography (CT). Plain radiographs demonstrated multiple diffuse radiopaque calcified deposits within the soft tissues (Figure 4). Whole-body CT confirmed extensive subcutaneous and periarticular calcifications without bone destruction, visceral involvement, lymphadenopathy, or underlying neoplastic lesions (Figure 5). Thyroid and parathyroid ultrasonography findings were normal. Based on the histopathological findings, normal phosphocalcic metabolism, negative immunological investigations, and exclusion of connective tissue diseases, metabolic disorders, malignancy, infectious etiologies, and iatrogenic causes, a diagnosis of idiopathic calcinosis cutis universalis was made. 

**Figure 6:**
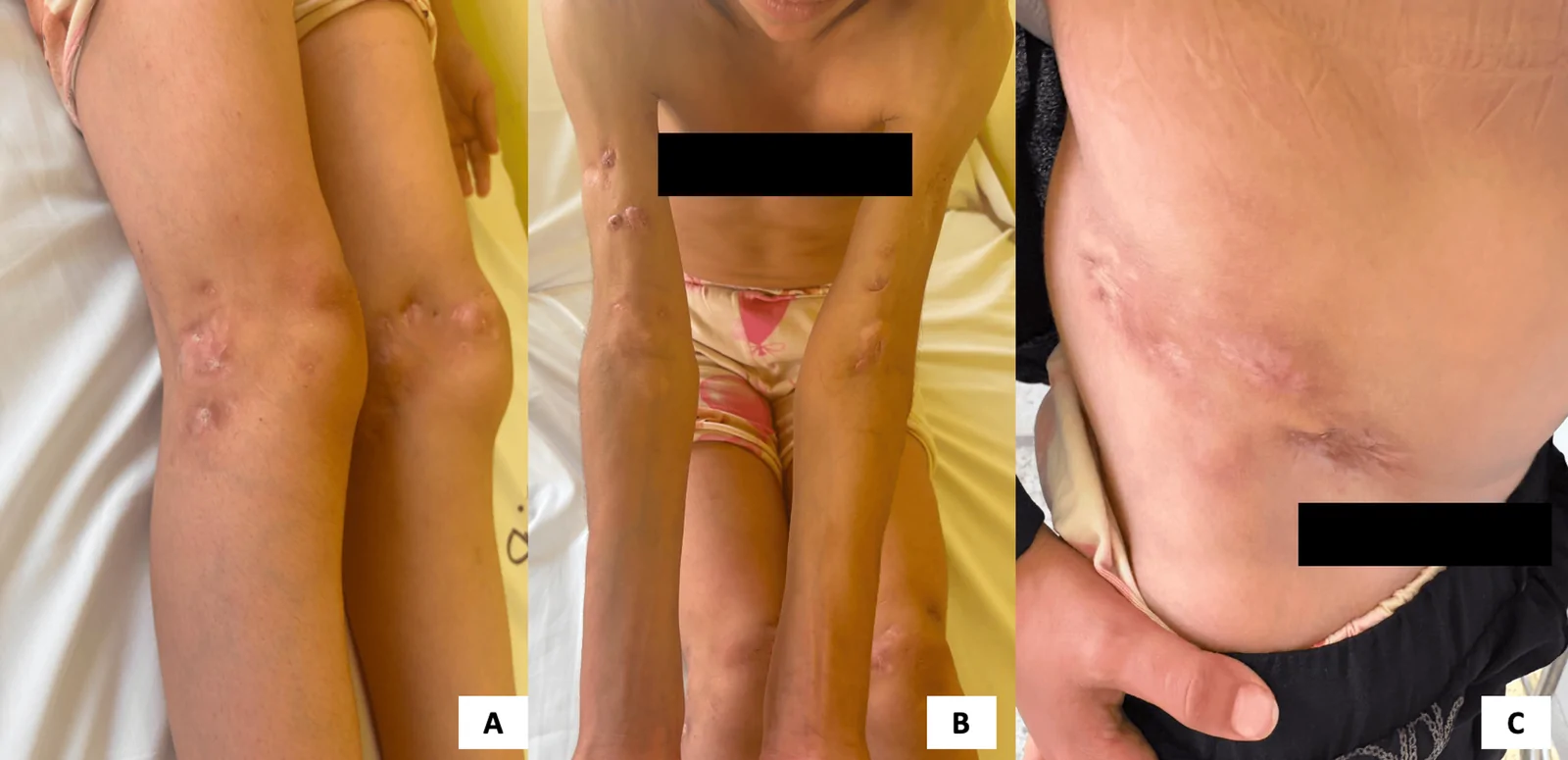
Clinical images after one year of treatment. Images obtained after one year of treatment showing marked regression of the cutaneous and subcutaneous calcinosis lesions with residual scarring and restoration of near-normal skin appearance. (A) Thighs. (B) Upper limbs. (C) Inguinal region.

**Figure 7:**
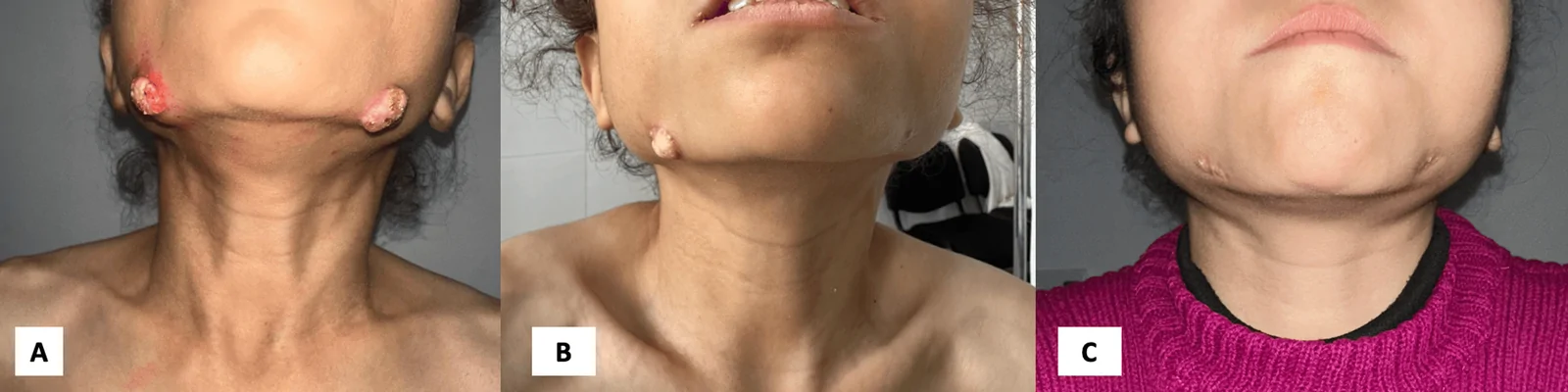
Clinical response to topical sodium thiosulfate therapy. (A) Initial clinical presentation showing bilateral mandibular calcinosis cutis lesions. (B) After three months of topical sodium thiosulfate application, partial regression was observed, with complete improvement of one lesion and approximately 50% reduction of the contralateral lesion. (C) After six months of treatment, complete clinical regression of both mandibular calcinosis lesions was observed.

Given the extensive disease burden and inflammatory features, treatment consisted of oral corticosteroids (2 mg/kg/day), progressively tapered to a maintenance dose of 5 mg/day. Intravenous zoledronic acid (0.05 mg/kg, diluted in 100 mL of 0.9% saline and infused over 30 minutes) was administered every three months for a total of six cycles. Because juvenile systemic sclerosis could not be completely excluded during the initial diagnostic assessment despite the absence of specific clinical or immunological findings, a calcium channel blocker (nicardipine, prolonged-release formulation) was initiated to prevent potential vasospastic manifestations and was subsequently replaced with diltiazem (60 mg/day). Vitamin D supplementation was administered to correct the deficiency. Topical sodium thiosulfate, prepared in a cold cream-based water-in-oil emulsion, was applied twice daily to ulcerated and fistulized lesions, together with appropriate local wound care. Extracorporeal shockwave therapy was performed for symptomatic bulky calcified lesions, while surgical excision was reserved for selected lesions causing persistent functional impairment (Figure 6). Of note, topical sodium thiosulfate was initially applied only to the mandibular lesions before being extended to other sites; faster regression of these facial lesions was observed compared with the untreated lesions elsewhere on the body during the same period, suggesting a specific local effect of the treatment (Figure 7).

**Figure 8:**
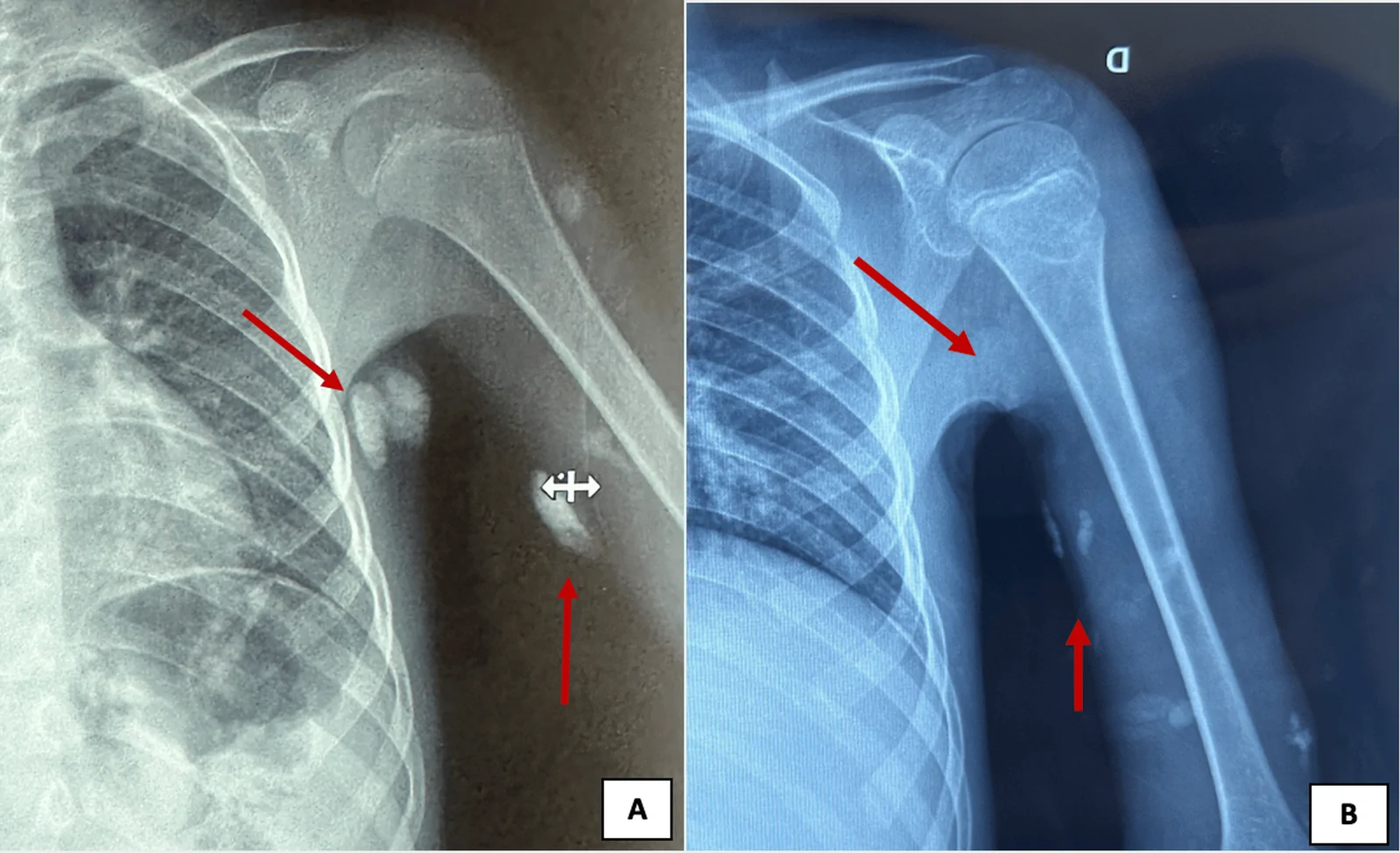
Radiological improvement after treatment. (A) Plain radiograph of the upper limb before treatment showing extensive radiopaque calcified deposits within the soft tissues of the arm (red arrows). (B) Follow-up radiograph after six months of treatment demonstrating marked regression of the calcified deposits (red arrows).

The patient was followed every three months for a total of 36 months, with regular clinical assessment, serial monitoring of phosphocalcic parameters, repeated immunological evaluation (antinuclear antibodies, anti-dsDNA, and the myositis-specific antibody panel), and radiological assessment of calcified lesions (Figure 8). Progressive radiological regression of the calcified deposits was observed following zoledronic acid therapy and extracorporeal shockwave treatment. Complete healing of the ulcerated and fistulized lesions was achieved after topical sodium thiosulfate therapy, with complete re-epithelialization and disappearance of calcium extrusion. Clinically, pain markedly decreased, normal walking ability was restored, and functional impairment resolved. During follow-up, no clinical or immunological evidence of an underlying connective tissue disease emerged, and phosphocalcic parameters remained within normal limits. 

## Discussion

Calcinosis cutis refers to the abnormal deposition of calcium salts, mainly calcium phosphate crystals, within the skin and subcutaneous tissues and may extend to deeper structures such as fascia, muscles, tendons, and occasionally visceral organs. It is not a disease entity itself but rather a cutaneous manifestation shared by several disorders, traditionally classified as dystrophic, metastatic, iatrogenic, idiopathic, or calciphylaxis [1]. The term “universalis” describes diffuse and extensive involvement, in contrast to the localized form known as calcinosis circumscripta.

In children, calcinosis cutis is uncommon and is most frequently dystrophic, occurring in the setting of connective tissue diseases. Juvenile dermatomyositis (JDM) represents the main associated disorder, with calcinosis reported in approximately 20% to 75% of patients, particularly in severe, prolonged, or inadequately controlled disease. Calcinosis may occasionally precede the diagnosis of JDM and represent its initial manifestation [2]. Idiopathic forms have rarely been described in childhood [3], and pediatric series have shown that autoimmune connective tissue diseases account for the majority of cases, whereas idiopathic forms remain exceptional [4]. Severe JDM-related calcinosis may be extensive, ulcerated, periarticular, and responsible for major functional impairment, including joint contractures [5]. Systemic sclerosis represents a much rarer but recognized association, generally accompanied by other characteristic clinical features such as Raynaud phenomenon or cutaneous sclerosis [6].

Because idiopathic calcinosis cutis universalis remains a diagnosis of exclusion, a broad differential diagnosis was carefully considered in our patient. Juvenile dermatomyositis, including its amyopathic form, was considered, given the marked inflammatory syndrome and mildly elevated lactate dehydrogenase; however, it was deemed unlikely because of the absence of proximal muscle weakness, normal creatine kinase, the absence of characteristic cutaneous signs (heliotrope rash and Gottron papules), and a negative comprehensive myositis-specific antibody panel. Juvenile systemic sclerosis was also considered but was not supported by the absence of Raynaud phenomenon, cutaneous sclerosis, or digital ulcers or by negative anti-centromere and anti-Scl-70 antibodies. Nonetheless, because a mild restrictive ventilatory pattern was noted, this diagnosis could not be formally excluded at presentation and justified prolonged surveillance. Tumoral calcinosis and hyperphosphatemic familial tumoral calcinosis were excluded by the normal serum phosphorus and calcium-phosphate product and the absence of a family history, since these entities are typically associated with hyperphosphatemia. Finally, inherited disorders of ectopic calcification were considered unlikely given the normal phosphocalcic metabolism and the absence of a suggestive family history or associated syndromic features. The convergence of these negative findings supported the diagnosis of idiopathic calcinosis cutis universalis.

This diagnosis nevertheless requires prolonged follow-up, as calcinosis may occasionally represent the first manifestation of an evolving connective tissue disease, particularly juvenile dermatomyositis [2]. In our patient, the marked inflammatory syndrome and mild restrictive pattern on pulmonary function testing, although nonspecific, justified regular clinical, biological, immunological, and functional surveillance over 36 months, during which no underlying autoimmune disorder emerged.

Treatment of idiopathic calcinosis cutis remains challenging, as no standardized therapeutic protocol exists. Management is individualized according to lesion extent, pain, functional consequences, and the presence of ulceration or infection. Reported therapeutic options include bisphosphonates, calcium channel blockers such as diltiazem, surgical excision, extracorporeal shockwave therapy, and sodium thiosulfate, although most evidence comes from case reports and small series [7]. Bisphosphonates are thought to act by reducing calcium turnover, inhibiting macrophage pro-inflammatory activity, and interfering with the formation and deposition of hydroxyapatite crystals.

Sodium thiosulfate has gained increasing interest because of its calcium-chelating properties: it forms soluble calcium thiosulfate complexes, thereby increasing the solubility of calcium deposits, and also exerts antioxidant and vasodilatory effects. While intravenous administration has mainly been reported in calciphylaxis and extensive calcifications [8,9], topical formulations have emerged as an attractive alternative because of their local effect and favorable safety profile. Topical sodium thiosulfate at concentrations ranging from 10% to 25% has been associated with progressive regression of calcified lesions, reduction of pain, and extrusion of chalky calcium material through ulcerated areas [10-12]. The preparation of a water-in-oil emulsion using a cold cream base has been described as improving local penetration and formulation stability, with clinical improvement reported in pediatric calcinosis cutis [11].

In our patient, topical sodium thiosulfate applied twice daily resulted in complete regression of fistulized calcinosis lesions, disappearance of chalky discharge, and healing of cutaneous lesions. Notably, because it was initially applied only to the mandibular lesions, a faster local response was observed at that site than elsewhere, suggesting a specific effect of topical sodium thiosulfate. In addition, zoledronic acid and extracorporeal shockwave treatment contributed to significant regression of deeper calcifications, restoration of walking ability, and improvement in quality of life. This case highlights the potential benefit of a multimodal approach combining systemic and local therapies in severe pediatric idiopathic calcinosis cutis universalis.

This report has several limitations. It is based on a single case, which limits the generalizability of the findings. Because several therapies were administered concurrently, the individual contribution of each treatment to the overall clinical improvement cannot be reliably determined, and a causal relationship between any single intervention and the observed outcome cannot be firmly established, with the partial exception of the facial lesions initially treated with topical sodium thiosulfate alone. Finally, although the 36-month follow-up is reassuring, longer surveillance remains necessary, as an underlying connective tissue disease may still declare itself later.

## Conclusions

Idiopathic calcinosis cutis universalis is an exceptionally rare cause of diffuse cutaneous calcification in children and remains a diagnosis of exclusion after careful investigation for connective tissue diseases, metabolic abnormalities, and other secondary causes. As calcinosis may precede the clinical manifestations of juvenile dermatomyositis or other autoimmune conditions, long-term clinical, biological, and immunological surveillance is essential, and the diagnosis of idiopathic calcinosis cutis should only be established after comprehensive exclusion of secondary causes and requires long-term clinical follow-up. Although no standardized treatment exists, a multimodal therapeutic approach combining medical and local therapies may provide significant clinical benefit. In our patient, the combination of zoledronic acid, extracorporeal shockwave treatment, and topical sodium thiosulfate resulted in marked clinical, functional, and radiological improvement, with healing of fistulized lesions and restoration of mobility. Early recognition and individualized management are crucial to limit functional impairment and improve quality of life.
